# Profound T Lymphocyte and DNA Repair Defect Characterizes Schimke Immuno-Osseous Dysplasia

**DOI:** 10.1007/s10875-024-01787-6

**Published:** 2024-08-17

**Authors:** Ondřej Vladyka, Jakub Zieg, Ondřej Pátek, Markéta Bloomfield, Zuzana Paračková, Anna Šedivá, Adam Klocperk

**Affiliations:** 1 Department of Immunology, 2nd Faculty of Medicine, Charles University and University Hospital in Motol, Prague, Czech Republic; 2 Department of Pediatrics, 2nd Faculty of Medicine, Charles University and University Hospital in Motol, Prague, Czech Republic; 3 Department of Internal Medicine, 2nd Faculty of Medicine, Charles University and University Hospital in Motol, Prague, Czech Republic

**Keywords:** Schimke immuno-osseous dysplasia, Schimke Imunoosseous dysplasia, Peritoneal dialysis, SIOD, Lymphopenia, Immunodeficiency, Thymus, Exhaustion, IL-7, RNA-seq, Spectral Cytometry, Cytometry, T cell

## Abstract

Schimke immuno-osseous dysplasia is a rare multisystemic disorder caused by biallelic loss of function of the *SMARCAL1* gene that plays a pivotal role in replication fork stabilization and thus DNA repair. Individuals affected from this disease suffer from disproportionate growth failure, steroid resistant nephrotic syndrome leading to renal failure and primary immunodeficiency mediated by T cell lymphopenia. With infectious complications being the leading cause of death in this disease, researching the nature of the immunodeficiency is crucial, particularly as the state is exacerbated by loss of antibodies due to nephrotic syndrome or immunosuppressive treatment. Building on previous findings that identified the loss of IL-7 receptor expression as a possible cause of the immunodeficiency and increased sensitivity to radiation-induced damage, we have employed spectral cytometry and multiplex RNA-sequencing to assess the phenotype and function of T cells ex-vivo and to study changes induced by in-vitro UV irradiation and reaction of cells to the presence of IL-7. Our findings highlight the mature phenotype of T cells with proinflammatory Th1 skew and signs of exhaustion and lack of response to IL-7. UV light irradiation caused a severe increase in the apoptosis of T cells, however the expression of the genes related to immune response and regulation remained surprisingly similar to healthy cells. Due to the disease’s rarity, more studies will be necessary for complete understanding of this unique immunodeficiency.

**Supplementary Information:**

The online version contains supplementary material available at 10.1007/s10875-024-01787-6.

## Introduction

Schimke immuno-osseous dysplasia (SIOD) is a very rare disease with a prevalence of 1:1–3,000,000, caused by the autosomal recessive mutations in the *SMARCAL1* gene, coding for a protein responsible for chromatin remodeling and DNA repair [1].

The phenotype of the disease is at least partly dependent on the residual activity of the protein [2], but patients are typically characterized by disproportionate growth failure due to spondyloepiphyseal dysplasia and nephrotic proteinuria, leading to kidney failure necessitating renal replacement therapy. Other symptoms may be present, including skin hyperpigmentations, refractory migraines, hypothyroidism or autoimmune diseases, mainly transient anemia or thrombocytopenia. [3–6].

Immunodeficiency is a prevalent and pronounced symptom of SIOD, affecting both cellular and humoral immunity and leading to the disease being classified as an inborn error of immunity according to the International Union of Immunological Societies (IUIS) classification published in 2022 [38]. Its hallmarks are severe hypogammaglobulinemia requiring immunoglobulin substitution, as well as impaired function of the thymus resulting in overall T cell lymphopenia [7]. T cells in SIOD have oligoclonal TCR repertoires with intrinsic defect of TCR signaling [8], are skewed towards memory stages, lack IL7Rα (CD127) and consequently have impaired responsiveness to IL-7 [9]. As *SMARCAL1* is expressed in the thymus [7] and works to limit DNA replication damage [10], we hypothesize that that VDJ recombination-induced DNA stress leads to thymic cell death in SIOD patients, resulting in low production of naïve T cells and their accelerated maturation, similarly to other DNA repair defect diseases such as Nijmegen breakage syndrome [11]. The primary immunodeficiency is further exacerbated by loss of immunoglobulins caused at least in part by the nephrotic syndrome and as a result, patients are highly susceptible to recurrent and sometimes life-threatening infections, which is particularly troublesome in the setting of continuous renal replacement therapy requiring the maintenance of peritoneal or venous catheters.

In this project, we aim to better characterize the immunodeficiency in SIOD patients, with particular focus on the functionality and phenotype of T cells, as well as the response to chromosomal damage on ex vivo primary patient samples.

## Methods

### Patients and Controls

Patients were recruited at the Department of Pediatrics and Department of Internal Medicine, 2nd Faculty of Medicine, Charles University and University Hospital in Motol. Corresponding healthy donors (HD) and patients undergoing peritoneal dialysis (PD) for non-immune-mediated kidney failure were used for comparison (individual details of patients and controls, including age and sex, are specified in Supplementary table S1). The study was approved by the Ethical Committee of the Motol University Hospital in Prague, Czech Republic (consent ID “EK-657/21”). All participants or their legal guardians signed an informed consent in accordance with the Declaration of Helsinki.

### Flow Cytometry

Peripheral blood from patients and HDs was collected into EDTA-coated tubes. For extracellular marker panels, 50 µl of full blood was immediately stained with antibody-fluorochrome conjugates for 15 min in the dark at room temperature, followed by red blood cell lysis with a NH_4_Cl-based lysis buffer for 15 min, centrifuged at 300 g for 5 min, resuspended in phosphate buffered saline (PBS) and measured on a BD LSRFortessa flow cytometer (BD Biosciences, San Diego, CA, USA). For panels including intracellular markers, peripheral blood mononuclear cells (PBMCs) were isolated and stained using previously published protocol [12]. The list of used antibody-fluorochrome conjugates is shown in Supplementary Table S2.

### Spectral Cytometry

Peripheral blood from patients and HDs was collected into EDTA coated tubes and PBMCs were obtained by Ficoll-paque density separation. The PMBCs were frozen in a medium containing 70% RPMI 1640 Medium, 20% fetal bovine serum (FBS) and 10% dimethyl sulfoxide (DMSO) using a MrFrosty container, then stored in liquid nitrogen until further batch-processing. After thawing, cells were cultured for 72 h in RPMI supplemented with 10% FBS, 1% PenStrep and 1% Glutamax, in the presence of IL-2, with or without the presence of IL-7 (both PeproTech, Thermo Fisher Scientific, Waltham, MA, USA). 400,000 PBMCs were cultured in 200 µl of media in a 48-well flat-bottom plate for each condition. For the last 6 h of culture the T-cells were stimulated by phorbol myristate acetate (PMA) and Ionomycin in concentrations of 50 ng/ml and 750 ng/ml, respectively. One hour after stimulation, Brefeldin A was added in concentration of 1 ng/ml. The cells were then washed with PBS, centrifuged at 300 g for 5 min, resuspended and stained with a mixture of antibody-fluorochrome conjugates (Supplementary Table S2) for extracellular markers and Brilliant Stain Buffer (BD Biosciences, San Diego, CA) for 15 min. After that, the cells were washed in PBS, fixed and permeabilized using the eBioscience FoxP3 FixPerm solution (ThermoFisher Scientific, Waltham, MA) according to manufacturer’s instructions and stained with a mixture of intracellular antibody-fluorochrome conjugates in eBioscience FOXP3 perm buffer for 30 min for intracellular staining. The cells were then centrifuged at 300 g for 5 min, resuspended in FluoroFix Buffer (BioLegend, San Diego, CA, USA) and measured on the CYTEK Aurora spectral cytometer (Cytek Biosciences, Fremont, CA, USA), the following day.

### γH2X Detection

Fresh whole blood was irradiated for with UV light for 10 min in 96-well NuncTM Cell-Culture Treated Multidishes (Thermo Fisher Scientific) to induce DNA breaks using the BIO-LINK BLX UV irradiation system (Vilber Lourmat, Collégien, France). After irradiation, the whole blood was incubated at 37 °C for 1 h and overnight. Following this, the whole blood was fixed with 4% formaldehyde (Thermo Fisher Scientific) for 10 min at 25 °C, erythrocytes were lysed using 0.1% Triton X-100 (Sigma Aldrich, Darmstadt, Germany) for 20 min at 37 °C, and leukocytes were permeabilized with 80% ice-cold methanol for 30 min. The samples were then labeled with antibodies against CD3 – Alexa 700 (clone MEM-57) (Exbio, Vestec, Czech Republic), and intracellular signaling was detected using anti-γH2X - PE antibody (BioLegend).

### Apoptosis Assay

Active caspase-3 and − 7 were detected using a FLICA caspase-3 and 7 assay kit (Thermo Fisher Scientific). PBMCs were irradiated for 10 min and left in the incubator for 1, 6–24 h. Then cells were incubated with the fluorescein-labeled inhibitor Z-YVAD-fmk (10 µM) for 1 h at 37 °C and CD3-A700 (clone MEM-57) (EXBIO)). The cells were washed three times and analyzed by flow cytometry with LSRFortessa flow cytometer (BD Biosciences)).

### RNA Sequencing

Fresh PBMCs of three patients and three HDs were irradiated for 10 min and then incubated for 6 hours at the concentration of 1 × 10^6^/ml. The RNA was then isolated and measured on nCounter SPRINT profiler machine with the use of Immunology v2 panel (NanoString Technologies, Inc., Seattle, WA), containing 581 gene probes and 15 housekeeping genes for normalization. PBMCs of patients P1, P3 and P4, as well as 3 samples from healthy donors, were measured for two conditions (UV irradiated and untreated).

### Data Analysis

For the experiments using flow cytometry, cell populations were manually gated using FlowJo (FlowJo LLC, Ashland, OR) and OMIQ softwares (Dotmatics, Boston, MA, USA). GraphPad Prism v10.1.2 (dtto) was used for statistical testing and visualization of the cytometry data.

Unless specified otherwise, p-values for the spectral cytometry data were obtained using 2-way ANOVA and corrected for multiple testing by Benjamini, Krieger and Yekutieli FDR method, with q value < 0.05 being considered significant. The NanoStringDiff v 1.32.0 package in R was used for normalization and subsequent analysis of the RNA seq data. The counts were normalized using housekeeping genes and two group comparisons were used to obtain the DEGs, defined as genes with adjusted p-value < 0.05 (Benjamini-Hochberg method). The data were then visualized in R with use of the Ggplot2 package.

## Results

### Immunodeficiency in SIOD Patients is Hallmarked by Profound T cell Lymphopenia

In this study, we explored the immune characteristics of 4 patients with genetically verified SIOD (3 male, 1 female, age at sampling 9.7 ± 5.65years) followed at the Department of Pediatrics and Department of Internal Medicine, Motol University Hospital in Prague, Czech Republic. Patient characteristics are shown in Table 1 and were discussed in detail in previous publications [3]. Patients presented at early childhood (2.3 ± 1.01years) with typical clinical features - disproportionate short stature, facial dysmorphism and steroid resistant proteinuric nephropathy. Average time to end-stage kidney disease (ESKD) was 1.5 ± 0.44years. Peritoneal dialysis was started in all children. Patient 1 died at the age of 20 years of cardiorespiratory failure as a result of progressive bacterial peritonitis and patient 2 died at the age of 4 years and 8 months of cardiorespiratory failure of uncertain etiology.

**Table 1:** – Clinical and routine laboratory data of the individual patients, normal ranges for laboratory parameters in round brackets

	P1	P2	P3	P4
Age at sample acquisition (years)	18.9	3.7	8.4	7.9
Sex	M	M	F	M
Mutation	NM_014140.4:c.1388G > A missense	NM_014140.4:c.2542G > T termination	NM_014140.4:c.1388G > A/ NM_014140.4:c.1000 C > T missense / termination	NM_014140.4:c.2542G > T termination
Age at manifestation (years)	4	1.5	1.6	2.1
Age at ESKD (years)	6	3.3	2.5	3.3
Complications	VZV, *Pseudomonas*,* Staphylococcus aureus* infections of catheter exit site	COVID 19, Protracted Adenovirus gastroenteritis	Recurrent headaches, transient ischemic attacks, protracted *influenza A* infection	Recurrent headaches, transient ischemic attacks, recurrent upper respiratory tract infections
Immune treatment	PD	PD, prophylactic ATB	PD, SCIG, prophylactic ATB	PD, SCIG, prophylactic ATB
IgG [g/l] **lowest; at the time of sampling**	↓ 6.33; 11.9 (6.37-11,05)	↓ 0,63; NA (5.5–10.2)	↓ 0,83; 7,84 (5.5–10.2)	↓ 0,89; 5,85 (5.5–10.2)
IgM [g/l]	∼ 0,94 (0,47 − 1,67)	∼ 0,99 (0,47 − 1,67)	∼ 1,4 (0,47 − 1,67)	∼ 0,72 (0,47 − 1,67)
IgA [g/l]	∼ 0,67 (0,58 − 1,16)	↓ 0,2 (0,33 − 0,91)	∼ 0,52 (0,33 − 0,91)	↓ 0,27 (0,33 − 0,91)
B cells [/µl]	∼ 200 (200–1500)	∼ 350 (200–2000)	↓ 90 (200–1500)	∼ 750 (400–3300)
B cell differentiation [%]	transitional ∼ 5.4 (0.9–5.7) naïve ∼ 70.42 (48.40–79.70) switched memory ↓ 3.04 (8.30–27.80) plasmablast ↓ 0.16 (0.40–2.40) marginal zone-like ∼ 19.22 (7.00-23.80)	NA	transitional ∼ 5.63 (4.60–8.30) naïve ∼ 53.71 (47.30–77.00) switched memory ∼ 17.88 (10.90–30.40) plasmablast ∼ 3,97 (0.60–5.30) marginal zone-like ∼ 20.26 (5.20–20.40)	transitional ↑ 12.51 (4.60–8.30) naïve ↑ 89.49 (47.30–77.00) switched memory ↓ 3.12 (10.90–30.40) plasmablasts ∼ 0.89 (0.60–5.30) marginal-zone-like ↓ 4.11(5.20–20.40)
T cells [/µl]	↓ 310 (1000–3900)	↓ 440 (1000–3900)	↓ 60 (1000–3900)	↓ 270 (1000–3900)
CD4 T cells [/µl]	↓ 170 (560–2700)	↓ 240 (900–2900)	↓ 40 (560–2700)	↓ 70 (560–2700)
CD8 T cells [/µl]	↓ 50 (300–1400)	↓ 190 (350–4200)	↓ 10 (300–1400)	↓ 90 (300–1400)
T cell proliferation	NA	normal	NA	NA

ATB = antibiotics; IS = immunosuppression; ESKD = end-stage kidney disease; NA = not available; PD = peritoneal dialysis; SCIG = subcutaneous immunoglobulin replacement; * age at the time of death

All patients had marked CD4 and CD8 T cell lymphopenia, which necessitated antibiotic prophylaxis with trimethoprim/sulfamethoxazole in 3 out of four patients. B cells were less affected, with a reduction of total B cell counts in only 1 out of 4 patients. However, immunoglobulin replacement was necessary in 2 patients due to stark reduction of serum IgG levels - notably, the eldest patient (P1) with only a discrete reduction of IgG bears a missense rather than a termination or compound missense/termination mutation. Parents of P2, whose IgG levels were severely decreased, did not approve SCIG therapy after adverse effects during the first dose. The development of B cells was affected in 2 out of 3 tested patients leading to a reduction in mature class-switched memory cell stages.

### SIOD T Cells are Skewed Towards a Memory Phenotype With a Strong Activation And Th1 Bias

To further investigate the T-cell phenotype beyond the low overall counts, we performed developmental T cell phenotyping in all patients using in vitro cell culture and spectral cytometry with functional readouts.

**Fig. 1:**
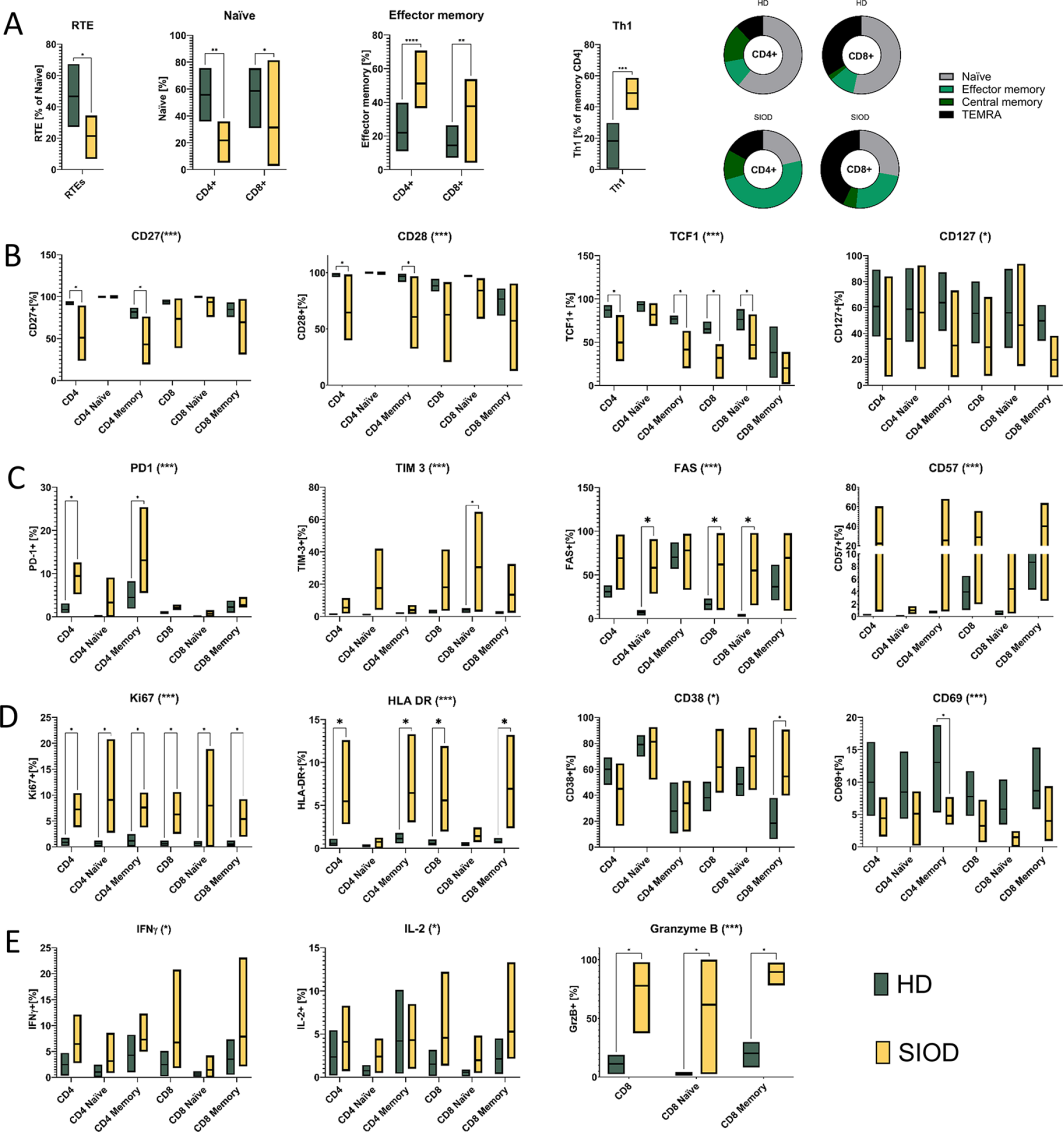
Memory populations and phenotype of SIOD patient T cells. RTE, naive, effector memory and Th1 T cells ex vivo (**A**). Expression of maturation (**B**), exhaustion/senescence (**C**), activation/proliferation (**D**) and functional readout markers (**E**) after 72 h culture. Y axis shows percentage of cells positive for a marker from population specified on X axis. Statistical significance was determined using two way ANOVA (significance for the “cohort“ variable indicated by asterisk in the plot title *p < 0.05, **p < 0.01 ***p < 0.001, ns = not significant) with multiple comparisons test corrected by Two-stage Benjamini, Krieger and Yekutieli method. FDR adjusted p-value of 0.05 was considered significant: *q < 0.05, **q < 0.01 ***q < 0.001

Therein, we observed significant differences characterized chiefly by a significantly decreased population of recent thymic emigrants (RTEs, CD4 + 31 + 45RA + 62 L+, Welch’s t-test *p* = 0.01) and mature naïve (CD45RA + 62 L+, 2-way ANOVA *p* = 0.004) T cell counts, with a relative increase of effector memory cells (CD45RA-62 L-, *p* < 0.0001) (Fig. 1A). Patient T cells displayed a significant loss of naïve and stemness markers CD27, CD28 and the transcription factor TCF1 (*p* = 0.0003, 0.0004 and < 0.0001, respectively) (Fig. 1B, Supplementary Fig. 2), and a concurrent increase of exhaustion-associated inhibitory receptors PD-1 (*p* = 0.0002) and Tim3 (*p* = 0.0005), the marker of cellular replicative senescence CD57 (*p* = 0.0004) and the pro-apoptotic receptor CD95/Fas (*p* > 0.0001) (Fig. 1C, Supplementary Fig. 2). The previously described decreased expression of CD127 [9], the surface receptor for IL-7 expressed mainly in naïve T cells, was also present in our data (*p* = 0.0119), but was mild and did not reach statistical significance in any individual T cell subset.

This highly differentiated phenotype was driven through increased proliferation measured by the expression of nuclear protein Ki-67 (*p* < 0.0001) (Fig. 1D, Supplementary Fig. 2). The persistent stimulation towards proliferation was reflected in T cell activation, with increased HLA-DR (*p* < 0.0001) and CD38 expression (*p* = 0.02), whereas the early activation marker CD69 was decreased (*p* < 0.0001).

Interestingly, within memory CD4 T cells we also observed a significant increase of the pro-inflammatory CXCR3 + CCR6- T helper 1 fraction (1-way ANOVA *p* < 0.0001) (Fig. 1E), and increased IFN-γ and IL-2 production by patient T cells (2-way ANOVA *p* = 0.0326, 0.0453, respectively). Similarly, cytotoxic CD8 T cells produced highly increased levels of granzyme B (*p* < 0.0001).

To exclude the effect of peritoneal dialysis on T cell maturation and activation we also analyzed 3 patients on peritoneal dialysis necessitated by other causes not associated with immunodeficiency and saw no difference in their T cell phenotype compared to healthy donors (Supplementary Fig. 1), suggesting that the reduction of naïve stages, upregulation of PD-1, activation and skew towards Th1 is an innate characteristic of SIOD.

Thus, we show that T cells in SIOD are fewer, skewed away from naïve and towards memory stages, with activated and exhausted phenotype and amplified proinflammatory features, especially with augmented production of IFN-γ and cytotoxicity.

### Interleukin-7 does not Cause Significant Difference in the T cell pool despite Lower CD127 Expression

**Fig. 2:**
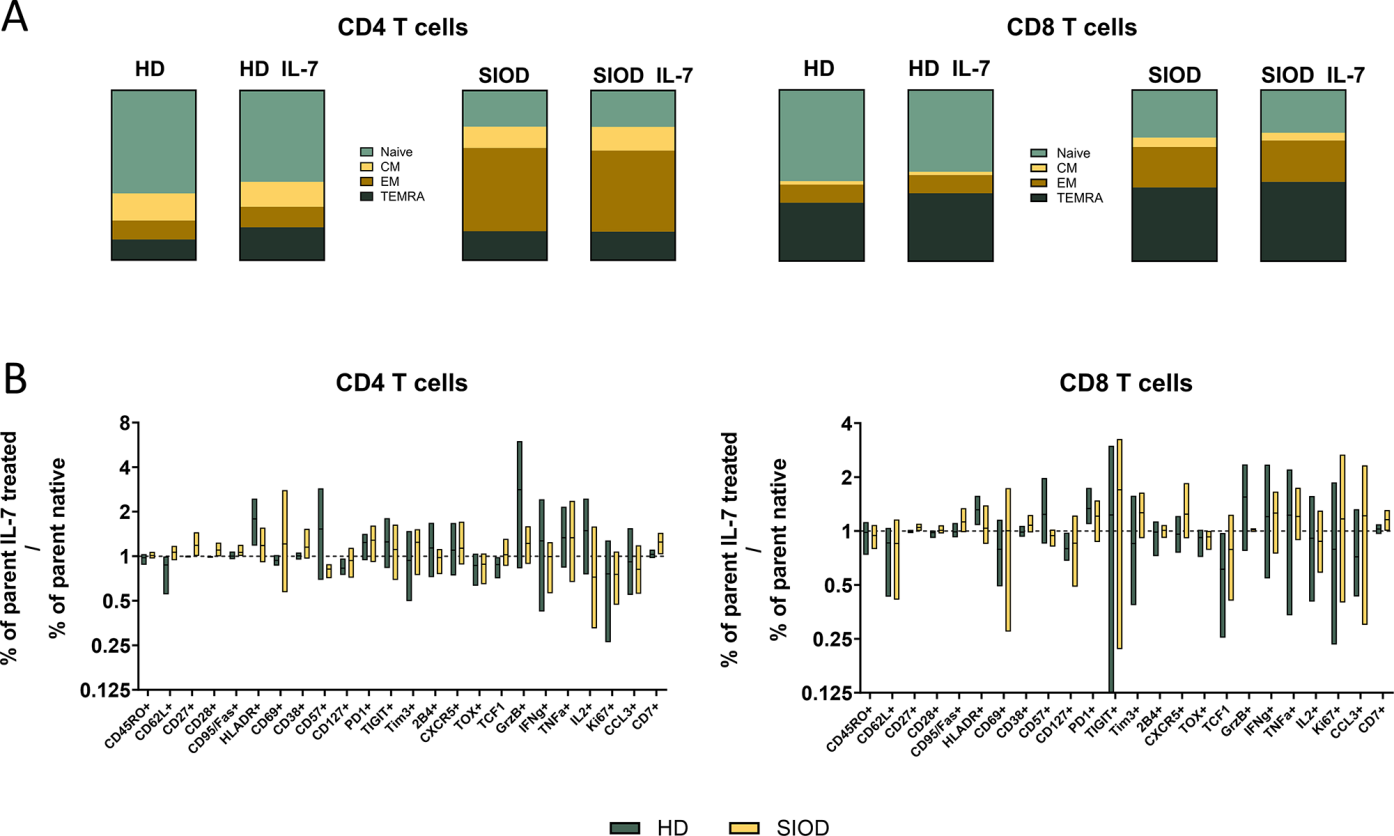
Effect of IL-7 on T cell phenotype. Proportion of naive, effector/central memory and TEMRA subpopulations from the CD4 and CD8 T cells respectively, in healthy controls and SIOD patients, for both IL-7 enriched and non-enriched culture media (**A**). Comparison of the CD4 and CD8 T cell phenotype in cells treated by IL-7, shown as log2 fold change of the percentage of cells positive for markers specified on the X axis compared to IL-7 non-treated cells (**B**)

To assess whether the T cells in SIOD respond differently to IL-7, we incubated HD and SIOD PBMCs for 72 h with/without 10ng/ml IL-7. In HD-derived T cells, IL-7 culture caused a slight but insignificant downregulation of CD127 (Fig. 2B) and a concomitant decrease of naïve T cells, with corresponding increase in the terminally differentiated TEMRA subpopulation (Fig. 2A). SIOD-derived T cells, on the other hand, failed to respond in similar fashion. No significant changes were observed in other phenotypic and functional features in either cohort (ratio paired t-tests for each marker native/IL-7 treated not significant) (Fig. 2B).

### SIOD T Cells are more Prone to Apoptosis and fail to Repair DNA Damage

The changes to T cell phenotype in SIOD patients suggest a significant impairment of physiological T cell development. As the SMARCAL1 protein seems to be associated with DNA damage repair [13, 14], we hypothesize that SIOD patient T cells may be susceptible to DNA damage. This, in turn, would result in T cell apoptosis during thymopoiesis, in particular in the VDJ recombination stages, ultimately leading to low thymic output and hyperactive homeostatic proliferation leading to premature T cell aging.

To test this theory, we irradiated fresh blood by UV light and assessed the amount of double-strand breaks (DSB) compared to HDs (26,58 ± 10,68 years) over time by measuring the presence of γH2X, a phosphorylated histone complex component which signals the presence of DBS to DNA repair proteins [15].

**Fig. 3:**
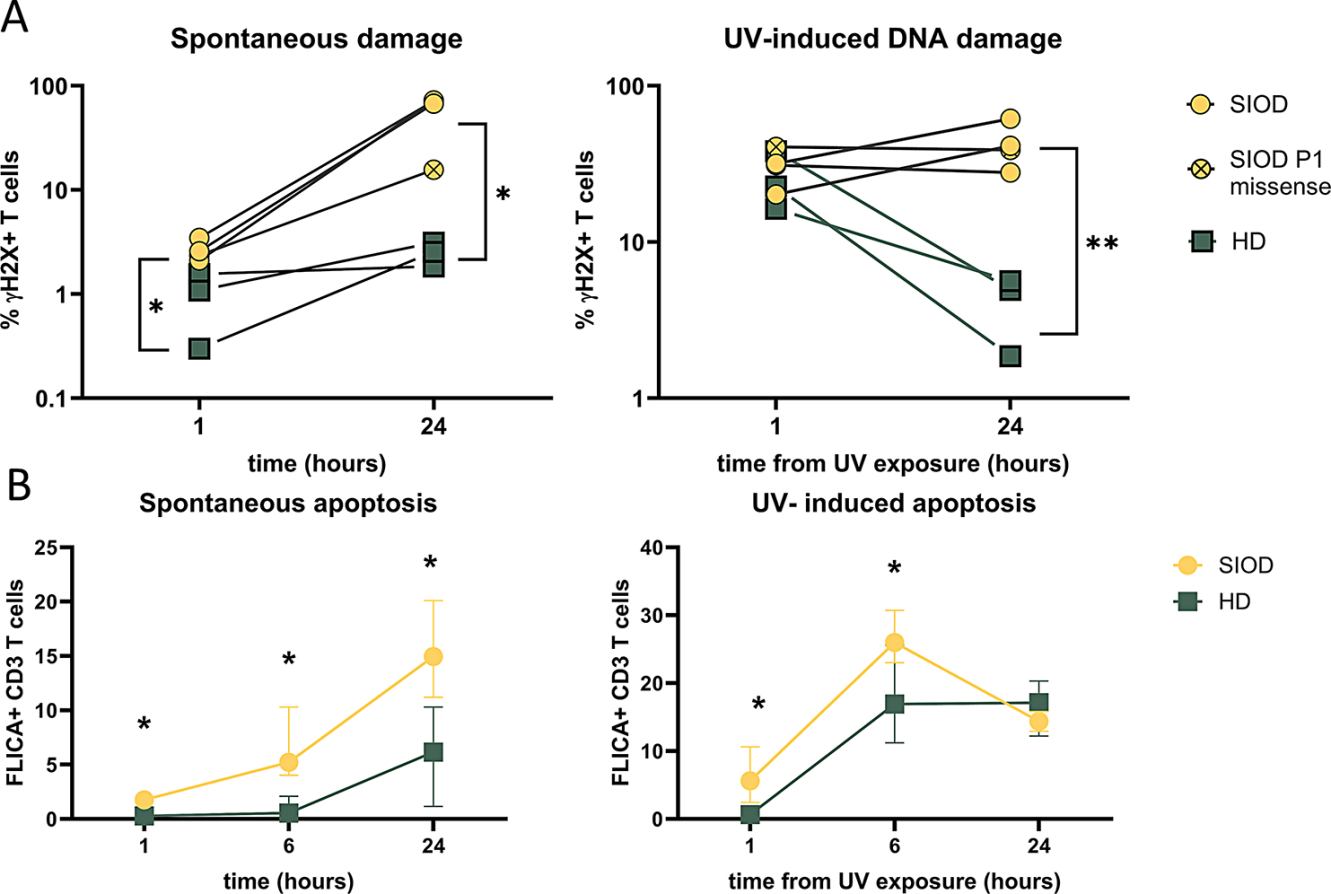
Response to DNA damage. Comparison of T cells positive for γH2X, indicator of double strand breaks, emerging spontaneously (left) or caused by UV induced DNA damage (**A**), after 1 and 24 h since intervention. Comparison of Fluorochrome-Labeled Inhibitors of Caspases (FLICA) positive T cells, measuring spontaneous or UV induced apoptosis (**B**), after 1, 6 and 24 h since intervention. Statistical significance was determined using unpaired t-test with multiple comparison corrected by Holm-Šídák method. *q < 0.05, **q < 0.01 ***q < 0.001

We found a significant increase in spontaneous DSB in SIOD patients (*p* = 0.0216) and a failure to repair the DSBs induced by UV irradiation (Fig. 3A). While both patients and controls had similar amounts of DSBs 1 h after irradiation, the HDs repaired the damage within 24 h while the SIOD patients failed to do so (*p* = 0.0063). This increased persistence of DNA damage resulted in increased apoptosis, where after 24 h an average of 15.3% of SIOD T cells were apoptotic, in contrast to 5.9% of cells in the HD group (p-value 0.0167) (Fig. 3B). The difference was exacerbated by UV irradiation with significant difference of apoptotic cells after 1 (*p* = 0.029) and 6 (*p* = 0.045) hours from UV irradiation.

### RNA Sequencing Reveals Preserved but Unique Transcriptomic Response to DNA Damage

**Fig. 4:**
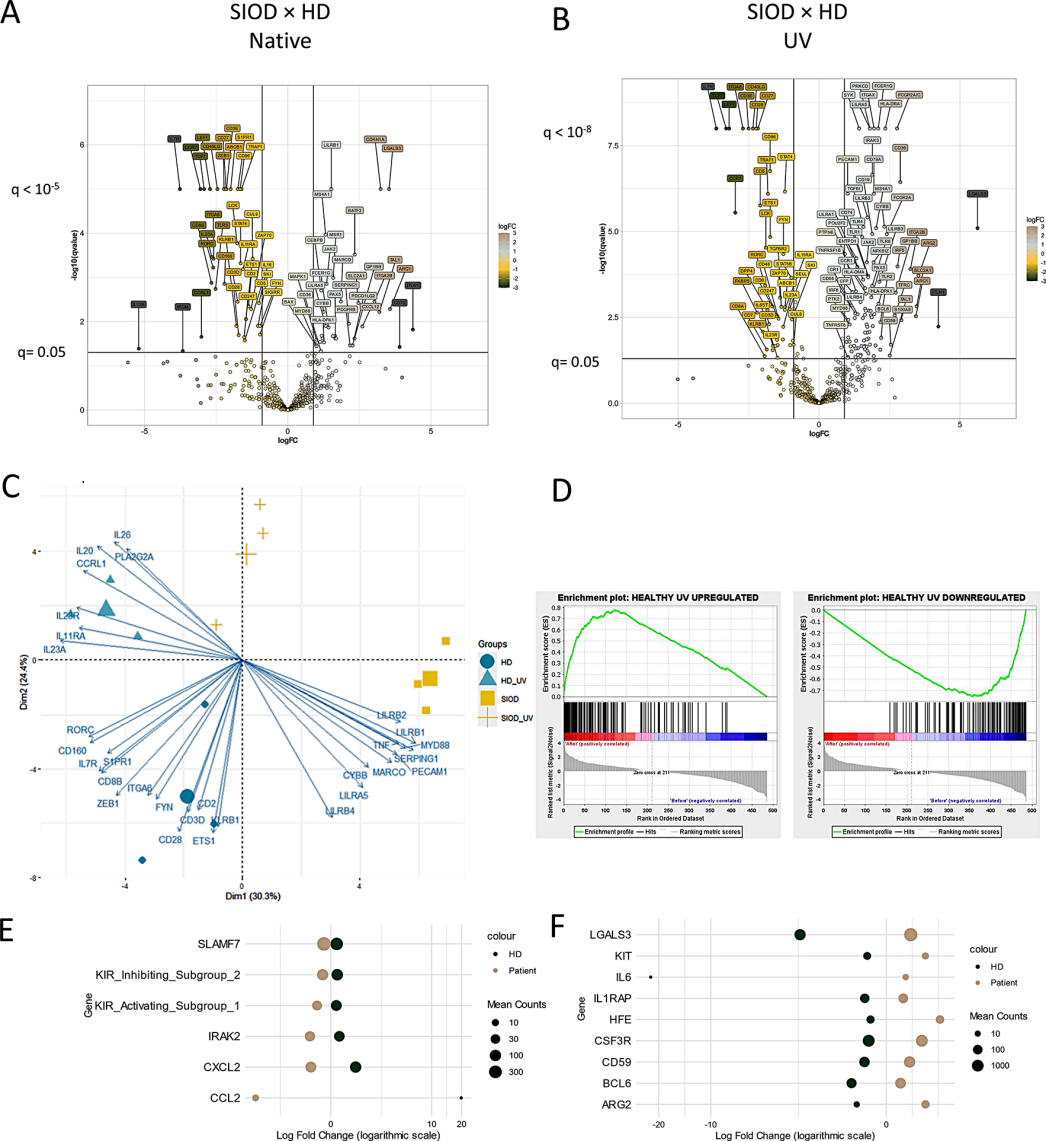
Transcriptomic features of SIOD PBMCs and their response to DNA damage. Volcano plots showing differential expression of genes in SIOD × HD PBMCs ex vivo (**A**) and after UV irradiation (**B**). Log2FC of genes is shown on X axis, positive values correspond to upregulation in SIOD. Y axis represents Benjamini-Hochberg FDR q-value as negative logarithm with significantly differentially expressed genes positioned above the horizontal line, capped at 10^− 5^ (for 4 A) or 10^− 8^ (for 4B). Principal Component Analysis of differentially expressed genes with 30 top contributing genes shown (**C**). Enrichment of gene sets containing genes up- or downregulated in HD after UV light irradiation, as expressed by irradiated SIOD PBMCs (**D**). Bubble plot of genes with opposite direction of differential expression after UV irradiation in HD and SIOD – upregulated in HD but downregulated in SIOD (**E**) or downregulated in HD but upregulated in SIOD (**F**)

The changes in T cell differentiation are rooted on transcriptional level. Assessing RNA expression in PBMCs using the 581 gene-wide nanoString Human Immunology kit, we noted 65 differentially expressed genes, including decreased expression of *IL7R*, *TCF7*, *CD27* and *CD5* (Fig. 4A), alongside increased expression of the anti-apoptotic *LGALS3*, and the costimulatory *CD70* and CD8 T cell memory-formation promoting *BATF3* [16]. Increased *TNF* mRNA expression in PBMCs yet unincreased TNF-α production by T cells in cell culture suggests that other cells, such as monocytes, might be another potent source of TNF-α in SIOD patients. With the exception of *TNF*, the transcriptomic analysis and flow cytometry produced equal results for the markers measured by both methods (Supplementary Fig. 3).

Principal component analysis (Fig. 4C) of all assessed genes shows a clear separation between the cohorts both in native state and after UV irradiation. As expected, genes with the strongest contribution to native HD cell phenotype include *IL7R* and *CD28*, hallmarks of T cell stemness and naivety. On the other hand, native SIOD cells are characterized by the expression of leukocyte immunoglobulin-like receptor subfamily genes, including *LILRA5*, which contributes to stimulation of TNF-α and other proinflammatory molecules production [17], *LILRB1* and *LILRB2*, which bind MHC-I and provide negative signals to T cells, thus inhibiting CD8 T cells [18], and *PECAM1* coding the adhesion molecule PECAM-1/CD31, another potent stimulator of TNF-α production in monocytes [19]. *TNF* itself was also strongly contributing to the native SIOD gene expression profile.

After UV irradiation, healthy cells upregulated 142 and downregulated 114 genes (Supplementary Figs. 4, 5 and 6). We used these genes to construct a “DNA-repair signature” and analyzed whether SIOD cells utilize similar transcriptomic changes to resolve UV-induced DNA damage. Indeed, SIOD cells strongly enriched the same signature after UV irradiation, suggesting that while some intrinsic differences between the groups persist, the response to DNA damage is largely similar (Fig. 4D). Despite the similar overall pattern (see also vectors in Fig. 4C), the number of genes expressed differentially between HD and SIOD increased from 65 to 154 after UV irradiation, including the novel upregulation of proinflammatory genes such as *IL6*, *S100A8*, *S100A9*, which were not differentially expressed in native cells (Fig. 4B).

We also identified 15 genes with opposite directions of response to UV irradiation between the two groups, i.e. genes upregulated by irradiated healthy cells yet downregulated by irradiated SIOD cells (Fig. 4E), or vice versa (Fig. 4F). Notably, SIOD PBMCs further upregulated (whereas HD PBMCs downregulated) a host of anti-apoptotic genes such as *LGALS3* (galectin 3) [20], *KIT*, *BCL6* or *ARG2*, possibly in an attempt to abrogate the DNA damage. Additionally, irradiated SIOD cells upregulated the expression of *IL1RAP*, which increases the (similarly increased) IL-6 production [21] and amplifies response to the (also increased) *KIT* oncogene [22].

Overall, transcriptomic analysis showed downregulation of genes related to T cell naivety and upregulation of genes associated with proinflammatory response, especially of innate immune cells. Patient cells responded in an orchestrated fashion to DNA damage, but interestingly, unlike cells derived from healthy donors, they responded with further augmentation of proinflammatory and anti-apoptotic genes.

## Discussion

The immunodeficiency in SIOD is a major cause of morbidity and mortality, with infectious complications being the leading cause of death [23]. While the observed hypogammaglobulinemia may be in part caused by profuse loss of antibodies into urine, IgG levels normalized only in one of our patients after attaining complete anuria. The origin of their characteristic T cell lymphopenia remains incompletely understood. Some evidence suggests a thymus-centric defect, supported by the fact that T cell signaling seems to be specifically affected in primary but not immortalized patient cells [8]. Further, healthy thymi express high levels of SMARCAL1, and SIOD patients were shown to have a restricted TCR repertoire and low in peripheral blood T-cell receptor excision circle (TREC) levels, correlating with disease severity [7]. At the same time, others have reported no change in size or structure of thymic tissue in SIOD patients [24] and since T cells themselves express *SMARCAL1* mRNA, an intrinsic T cell defect has also been proposed. The thymi of patients in this cohort were not available for study.

In this study, we employed spectral cytometry to assess the phenotype and function of T cells on single cell resolution. We recapitulated the previously described skew away from naïve and into memory T cells [9, 25], but also show loss of naïve/progenitor specific markers such as CD27 [26, 27], CD28 and TCF1 [28], as well as high proliferation status and acquisition of exhausted/senescent phenotype with high expression of PD-1, Tim3 and CD57. These findings are similar to those described by us and others in primary thymic disorders such as 22q22.1DS [29], where the lack of thymic production is partially offset through enhanced homeostatic proliferation in the periphery. Strikingly, another similarity between these two conditions is a strong skew towards the proinflammatory T helper 1 cell phenotype [30] producing IFN-γ. Such shift has also been described previously in homeostatically proliferated T cells after immunosuppression, which skewed towards Th1 phenotype and were highly proinflammatory [31]. Since oligoclonal TCR repertoire was reported in SIOD, peripheral expansion of T cells is likely also occurring in this disease [7]. Here, SIOD T cells produced higher levels of IFN-γ, IL-2 and granzyme B, regardless of the naïve/memory differentiation status. IFN-γ signaling has been shown to increase PD-1 L expression in a murine model of rejection-induced renal failure [32], which may contribute in some part to the IFN-γ/PD-1 feedback loop in SIOD patients. The effect of this inflammatory response on patient renal damage is unknown.

While CD127 expression was not uniformly decreased on patient cells in this study, in contrast to previous findings of Sanyal et al. [9], *IL7R* mRNA was dramatically reduced and patient cells failed to respond to IL-7-enriched culture. In contrast, IL-7 drove healthy donor-derived cells towards memory phenotype, as it did previously for 22q11.2DS athymic patients [30] which also did not express lower levels of CD127 or *IL7R* mRNA. This supports the hypothesis that the lack of CD127 is a cell-intrinsic feature of SIOD T cells. Ideally, this could be tested by differentiating patient stem cells within artificial thymic organoids [33]. At present moment, however, IL-7 or other cellular immunomodulators do not seem to be suitable for the amelioration of SIOD T cell lymphopenia.

While the immunodeficiency caused by SIOD is treatable by hematopoietic stem cell transplantation (HSCT) [34], the patients suffer from transplantation related complications with a very high mortality rate, possibly due to increased sensitivity to the genotoxic drugs administered during the treatment [35, 36]. This work supports previous reports of SMARCAL1 involvement in DNA repair [10, 37] by demonstrating the reduced rate of recovery in primary patient lymphocytes after UV irradiation and an increased spontaneous and UV-induced apoptosis. Other DNA-repair defects are known to cause profound T cell lymphopenia, including Nijmegen breakage syndrome, ataxia telangiectasia, Artemis or DNA ligase IV deficiency, and others [38]. We additionally show here that SIOD leukocytes respond to UV irradiation similarly to healthy cells on mRNA level, but with increased innate inflammatory response and upregulation of anti-apoptotic genes. As the functional assay clearly showed increased UV-induced apoptosis, we propose that the upregulation of anti-apoptotic genes is an attempt to stall this process, which nevertheless fails due to impaired DNA-repair. This result implies that patients should be protected from excessive radiation during imaging studies, similarly to those with other DNA repair disorders [39], especially as several cases of non-Hodgkin lymphoma and other malignancies have been reported in SIOD patients [36, 40].

As a secondary result, we found that in two out of three patients tested, B cell maturation into class-switched memory B cells was impaired, with an increase of transitional B cells in one but not the other two patients. Bertulli et al. found the increase in transitional cells to be more characteristic and did not observe class-switching to be affected [25], however, their patients carried a different missense rather than termination mutation. Despite all patients receiving peritoneal dialysis, the patient with mildest immunologic phenotype bearing only single missense mutation (P1) required least immunoglobulin substitution, suggesting lack of production rather than purely loss-based etiology of the hypogammaglobulinemia.

The main limitation of our work is the low number of patients with this disease, stemming from its rarity, which, along with some degree of variability of the phenotype of T cells, rendered some of the differences between cell subsets statistically insignificant, despite substantial difference in multivariate expression of the markers. Further, the access to biological material was limited due to poor clinical state of patients, deep T cell lymphopenia and poor venous access, as well as two patient deaths during the course of the study. Further investigations using artificial thymic organoid assays could resolve the thymic/cell-intrinsic defect debate, and single-cell RNA sequencing could pinpoint the inflammatory changes into specific cell types, such as monocytes/M1 macrophages, or Th1 cells.

In summary, here we show in 4 patients with SIOD a chronically activated and exhausted T lymphocyte state accompanied by a memory, Th1 and cytotoxic skew, as well as impaired differentiation of B cells. Crucially, we also document poor response to DNA damage, with increased apoptosis and inflammatory response after UV-irradiation. Our findings support the paradigm of cell-intrinsic cell defect in SIOD, curable by HSCT, but caution against the use of DNA-damaging medication and procedures.

## Electronic Supplementary Material

Below is the link to the electronic supplementary material.

Supplementary Material 1

## Data Availability

The data that support the findings of this study are available from the corresponding author upon request.
