# Supplementary material for: Profound T Lymphocyte and DNA Repair Defect Characterizes Schimke Immuno-Osseous Dysplasia
Source: J Clin Immunol. 2024 Aug 17;44(8):180. doi: 10.1007/s10875-024-01787-6 (PMC11330395; doi:10.1007/s10875-024-01787-6)
Supplement: Supplementary file 1 — Supplementary Material 1 [file 10875_2024_1787_MOESM1_ESM.pdf]

ID	Cohort	Sex	Age at the time of sample collection	Diagnosis leading to PD	Average age	Used for spectral cytometry	Average age (spectral cytometry)
1	HD	M	16.1		13.26667		12.3
2	HD	M	11.7			YES	
3	HD	F	11.5			YES	
4	HD	M	14.6				
5	HD	F	16				
6	HD	F	11.6				
7	HD	F	13.6				
8	HD	F	13.3				
9	HD	M	17.2				
10	HD	F	11.5				
11	HD	M	14				
12	HD	F	11				
13	HD	M	13.1			YES	
14	HD	M	12.9			YES	
15	HD	M	10.9				
1	SIOD	M	18.9	SIOD	9.725	YES	9.725
2	SIOD	M	3.7			YES	
3	SIOD	F	8.4			YES	
4	SIOD	M	7.9			YES	
1	PD	F	17.5	Hemolytic-uremic syndrome	14.46667		
2	PD	M	18.5	Nephrotic syndrome, steroid resistant			
3	PD	F	7.4	Congenital nephrotic syndrome, NPHS1 mutation			
1	HD - nanoString	F	20.3		26.575		
2	HD - nanoString	F	12.5				
3	HD - nanoString	F	39.2				
4	HD - nanoString	M	34.3				

Supplementary table S1: Cohort information

Fluorochrome	Antigen	Clone	Host species	Dilution
BUV395	CD3	UCHT-1	mouse	1:50
Live/Dead Blue	Viability			1:100
BUV496	CD69	FN50	mouse	1:50
BUV615	CD27	L128	mouse	1:50
BUV661	CD62L	DREG-56	mouse	1:50
BV421	2B4	2-69	mouse	1:25
BV480	CXCR5	RF8B2	rat	1:50
BV510	CD7	M-T701	mouse	1:50
BV570	CD45RO	UCHL1	mouse	1:50
BV605	TIGIT	A15153G	mouse	1:50
BV650	Tim-3	7D3	mouse	1:50
BV711	CD57	NK-1	mouse	1:500
BV785	PD-1	EH12.2H7	mouse	1:50
PE	CD95 (Fas)	DX2	mouse	1:10
PE-Cy5	CD28	CD28.2	mouse	1:50
PerCP	CD8	SK1	mouse	1:50
PerCP-Cy5.5	HLA-DR	L243	mouse	1:50
PerCP-eFluor710	CD39	A1	mouse	1:50
Spark NIR 685	CD127	A019D5	mouse	1:50
APC-H7	CD4	RPA-T4	mouse	1:50
APC-Fire 810	CD38	HIT2	mouse	1:50
BUV737	Ki-67	B56	mouse	1:500
BV750	IL-2	MQ1-17H12	rat	1:50
FITC	CCL3	CR3M	mouse	1:50
PE-eFluor610	TNF-a	MAb11	mouse	1:50
PE-Cy7	IFN-g	4S.B3	mouse	1:50
APC	TCF1	C63D9	rabbit	1:50
AF647	TOX	E6G5O	rabbit	1:50
R718	Grz B	GB11	mouse	1:50
AF488	CXCR5	J252D4	rat	1:100
PE	CXCR3	G025H7	mouse	1:50
BV605	CCR6	G034E3	mouse	1:50
BV421	PD1	EH12.2H7	mouse	1:50
AF700	CD3	MEM-57	mouse	1:100
BV650	CD4	RPA-T4	mouse	1:100
V500	CD8	RPA-T8	mouse	1:100
APC-H7	CD45RA	HI100	mouse	1:100
APC	CCR7	G043H7	mouse	1:50
BV605	CD27	L128	mouse	1:100
PerCP-Cy5.5	CD28	CD28.2	mouse	1:100
BV421	CD69	FN50	mouse	1:50
FITC	CD57	TB01	mouse	1:100
PE	Ki67	Ki-67	mouse	1:50

Supplementary table S2: Antibodies used

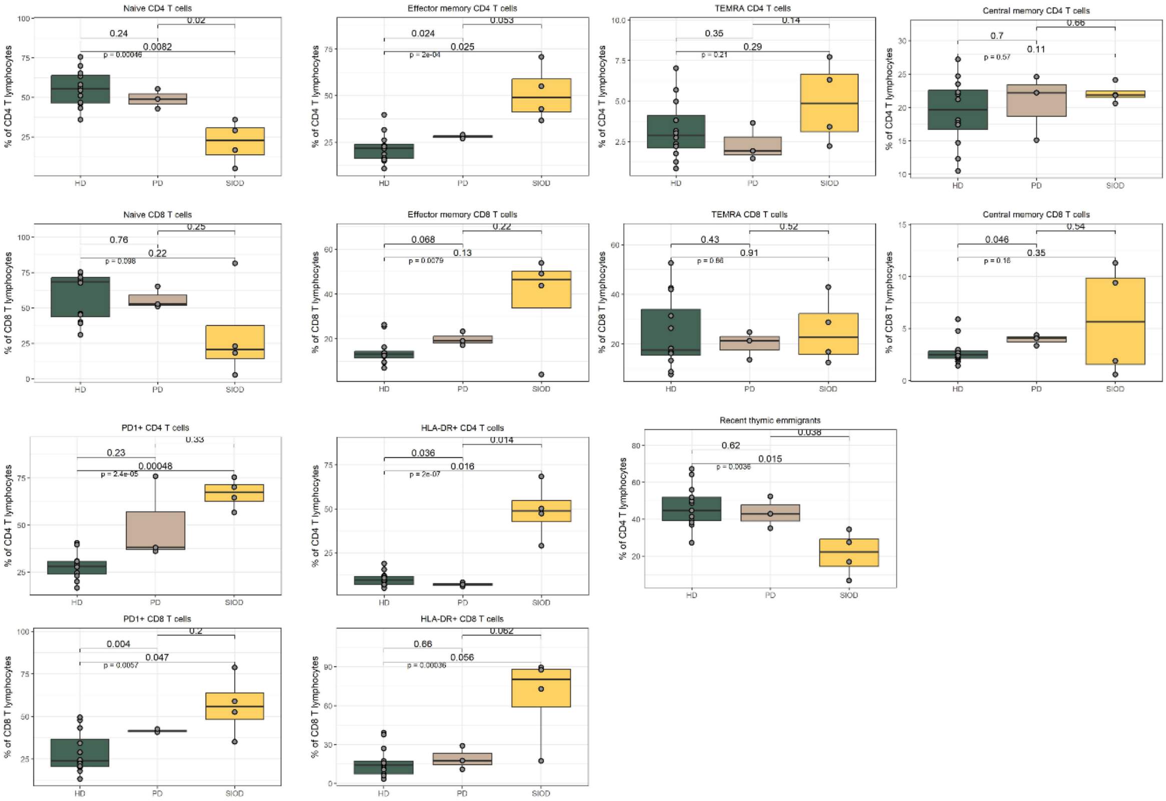

Supp. Fig. 1: Effect of peritoneal dialysis on T cell phenotype, boxplots show % of cells in gate defined in the respective plot titles. Naïve T cells were defined as $CD45RA^{+}CD62L^{+}$, Effector memory as $CD45RA^{-}CD62L^{-}$, Central memory as $CD45RA^{-}CD62L^{+}$, TEMRA cells as $CD45RA^{+}CD62L^{-}$. Recent thymic emigrants were defined as $CD4^{+}CD31^{+}$

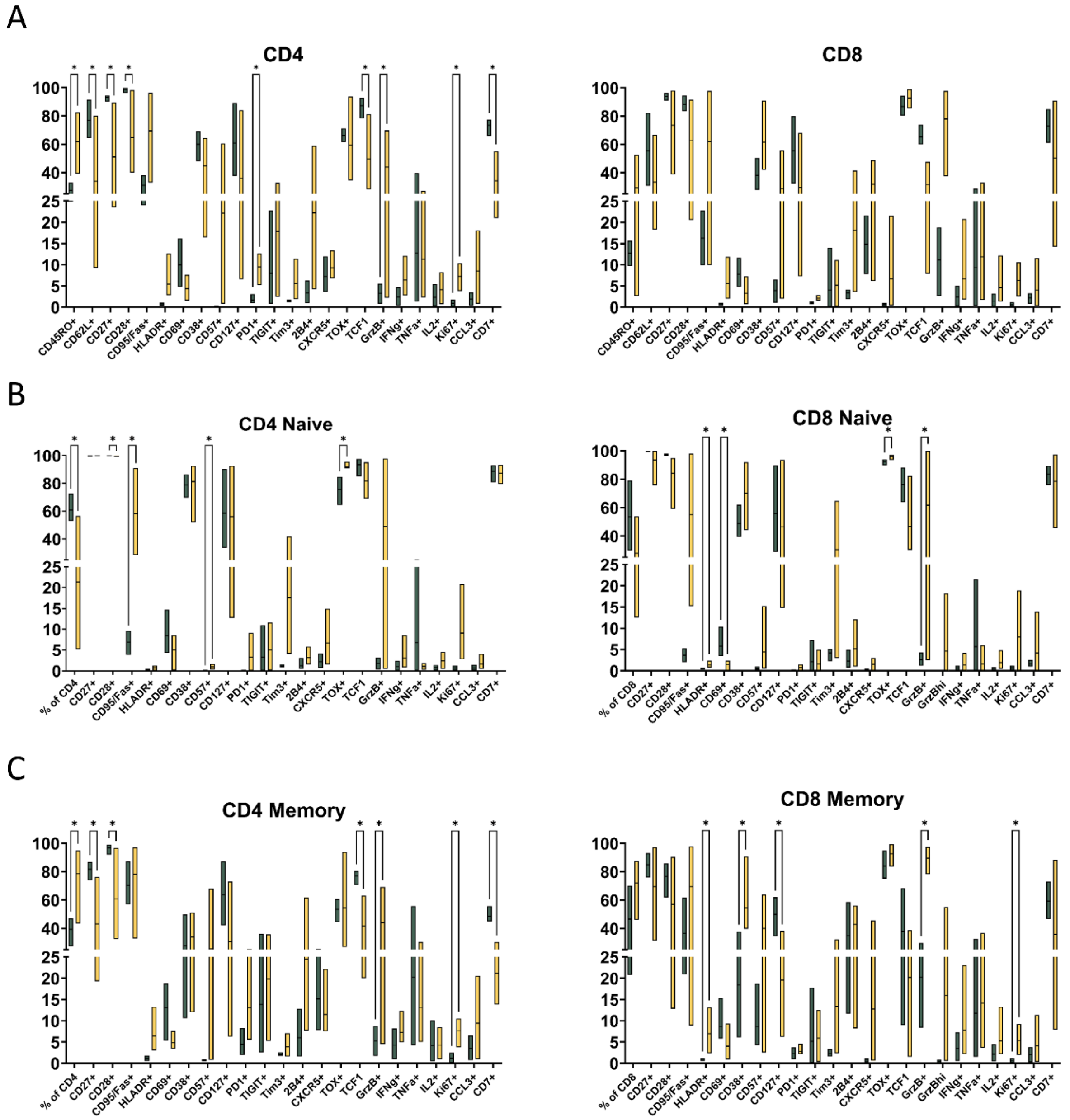

Supp. Fig. 2: Comparison of all markers measured in the spectral cytometry panel. Y axis shows percentage of cells positive for a marker specified on X axis. Statistical significance was determined by two-way ANOVA with multiple comparisons test corrected by Two-stage Benjamini, Krieger and Yekutieli method using GraphPad Prism v10.2. FDR adjusted p-value of 0.05 was considered significant. Fig 2A: shows whole T cell population, Naïve T cells were determined as CD45 RO$^-$ CD62L$^+$(Fig 2B), Memory T cells were defined as non-naïve T cells (Supp Fig2C.)

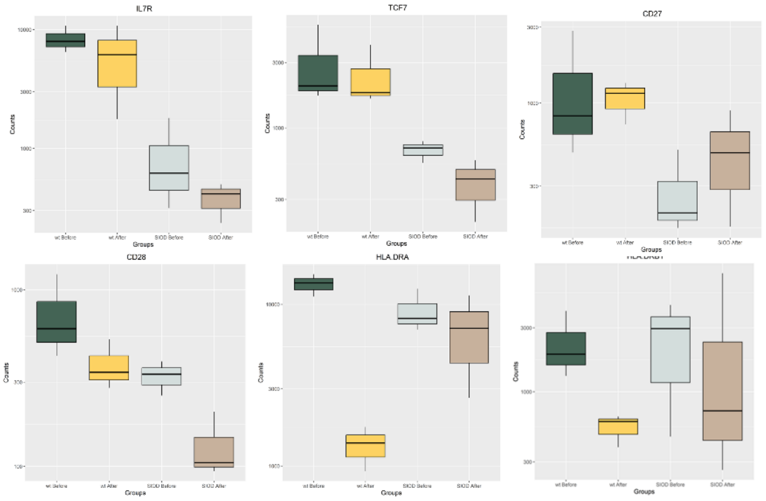

Supplementary Figure 3: mRNA expression of selected genes

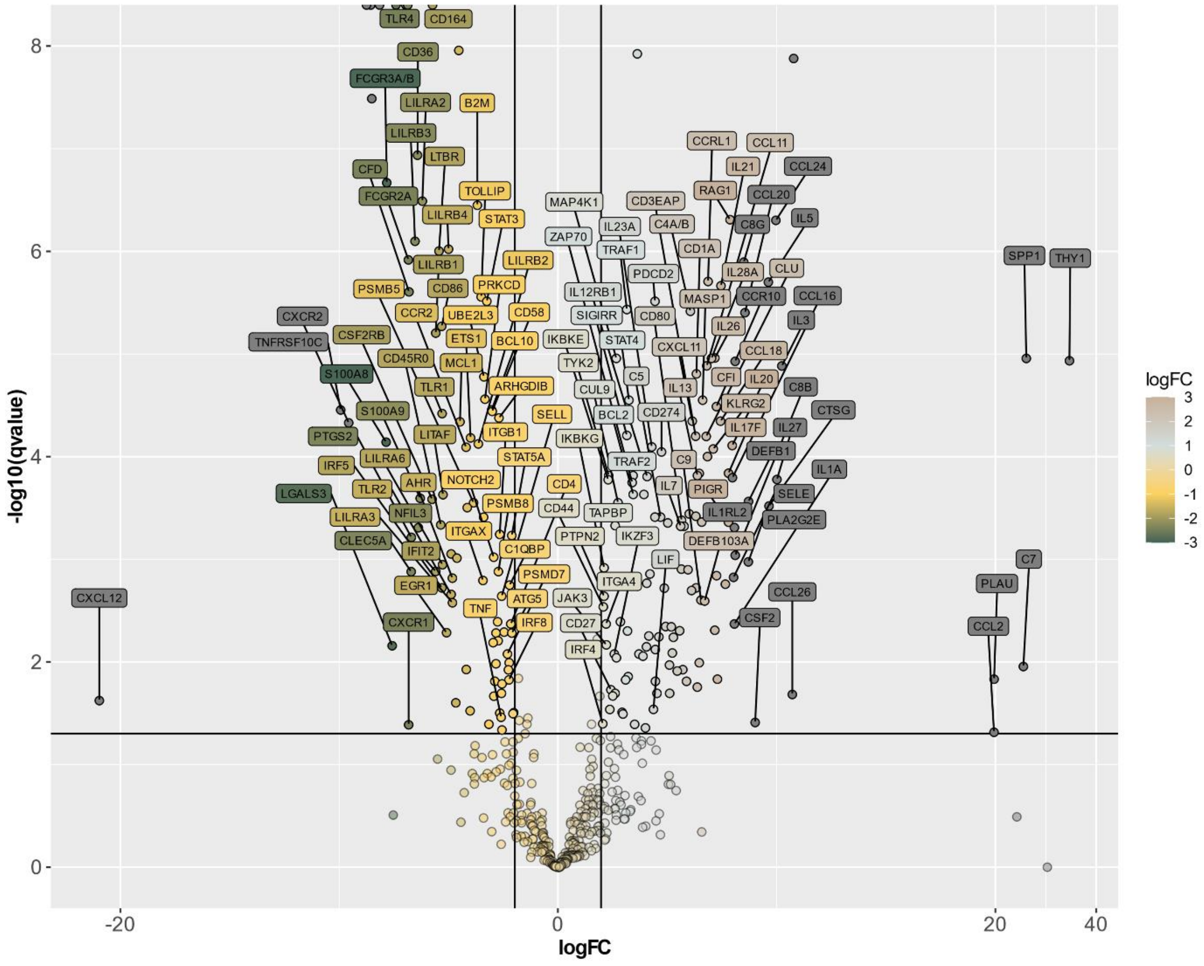

Supplementary Figure 4: DNA-repair signature

Supplementary Figure 5

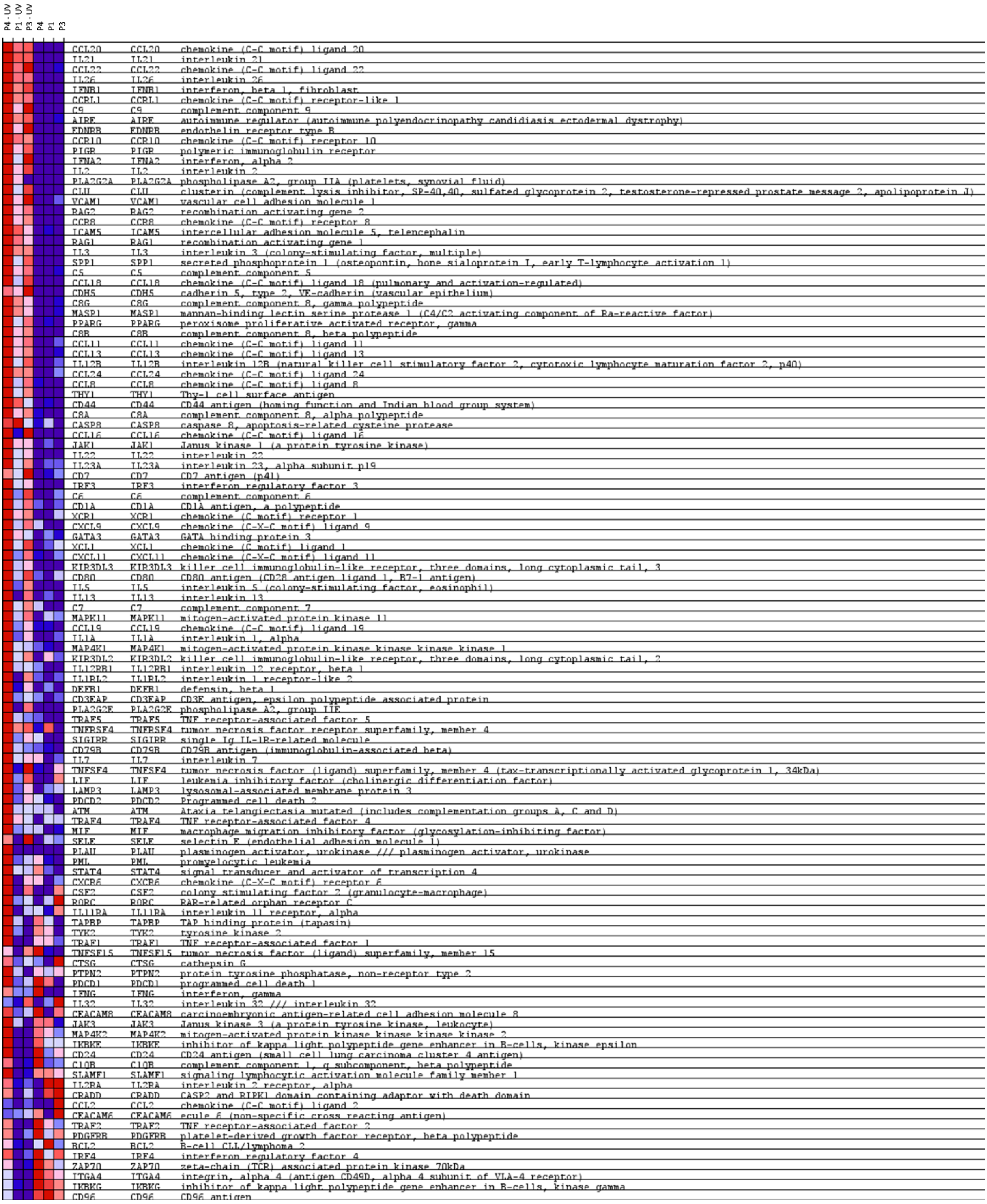

Supplementary Figure 5: Heatmap of expression of the geneset created from DEGs upregulated in HD after UV irradiation. Top 78 genes of the heatmap (all genes above Selectin E) have reached core enrichment also in the SIOD cohort.

Supplementary Figure 6

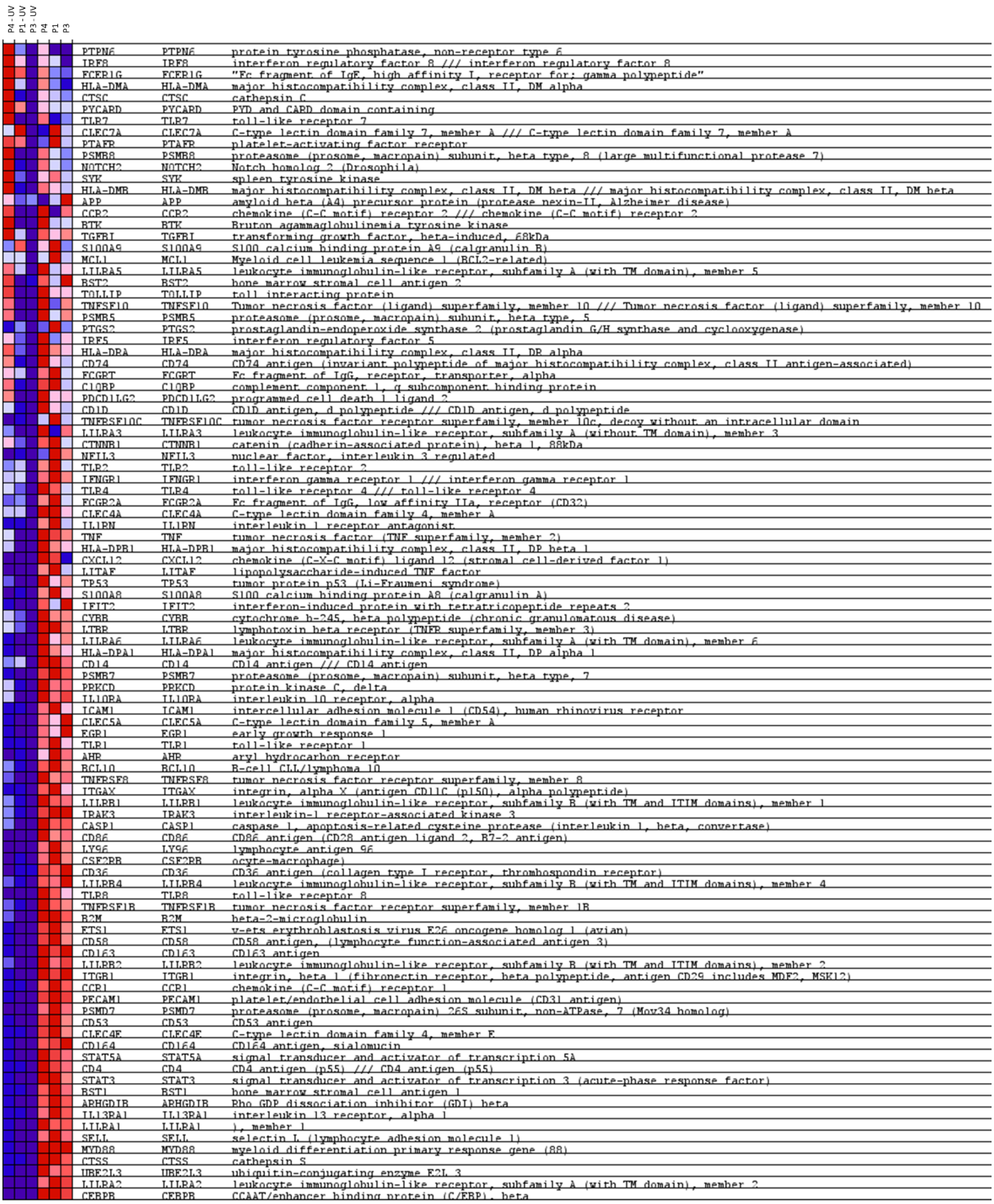

Supplementary Figure 6: Heatmap of expression of the geneset created from DEGs downregulated in HD after UV irradiation. Bottom 65 genes (all lines below LILRA3) also achieved negative core enrichment in SIOD cohort.
